# Ferric Uptake Regulator Contributes to *Pseudomonas donghuensis* HYS-Induced Iron Metabolic Disruption in *Caenorhabditis elegans*

**DOI:** 10.3390/microorganisms13051081

**Published:** 2025-05-06

**Authors:** Donghao Gao, Liwen Shen, Yelong Lin, Shuo Huang, Zhixiong Xie

**Affiliations:** Hubei Key Laboratory of Cell Homeostasis, College of Life Sciences, Wuhan University, Wuhan 430072, China; 2021202040036@whu.edu.cn (D.G.); 2023202040009@whu.edu.cn (L.S.); 2018302040100@whu.edu.cn (Y.L.); 2024202040016@whu.edu.cn (S.H.)

**Keywords:** *Pseudomonas donghuensis* HYS, *Caenorhabditis elegans*, ferric uptake regulator, iron metabolism, virulence factor

## Abstract

Iron is essential for vital biological processes, with its metabolism closely linked to host–pathogen interactions. *Pseudomonas donghuensis* HYS, with its superior iron uptake capacity, demonstrates pronounced virulence toward *Caenorhabditis elegans*. However, the virulence mechanisms remain unexplored. Ferric uptake regulator (Fur) regulates iron homeostasis and pathogenicity in bacteria, yet its role in HYS-mediated *C. elegans* pathogenesis requires systematic investigation. In this study, comparing the pathogenic processes of HYS and *P. aeruginosa* PA14 revealed that HYS causes stronger, irreversible toxicity via distinct mechanisms. Transcriptomics revealed that HYS infection disrupts *C. elegans* iron metabolism pathways, specifically iron transport, and iron–sulfur cluster utilization. Fur was identified as a pivotal regulator in HYS virulence and was indispensable for its colonization. Specifically, Fur was critical for disrupting nematode iron metabolism, as *fur* deletion eliminated this effect. While Fur regulated two HYS siderophores, neither of them mediated in the iron metabolism disruption of *C. elegans*. Screening identified Fur-regulated virulence factors to further investigate the function of Fur in HYS virulence, particularly alkaline proteases, and type II secretion system components. This study highlight that HYS can disrupt the iron metabolism pathway in *C. elegans*; Fur serves as a pivotal positive regulator in HYS-induced damage, particularly in disrupting iron metabolism through a siderophore-independent pathway. These findings expand the understanding of *Pseudomonas* pathogenicity and Fur-mediated virulence regulation.

## 1. Introduction

Iron is essential for life processes, serving as a critical component in numerous biochemical reactions and playing a pivotal role in physiological functions such as oxygen transport, electron transfer, and DNA synthesis [1]; therefore, the metabolic pathways responsible for iron absorption, transport, utilization, and storage play a vital role in organisms [2]. During the interaction between pathogens and hosts, pathogens frequently employ strategies that disrupt the host’s iron metabolic pathways to circumvent “nutritional immunity”, thereby facilitating their pathogenicity [1,3].

*Pseudomonas* are environmentally widespread microorganisms renowned for their strong adaptability, including pathogenic species that affect plants and animals, such as the human opportunistic pathogen *P. aeruginosa* [4], the fish pathogen *P. putida* [5], and the plant pathogen *P. syringae* [6]. *P. aeruginosa* PAO1 can cause severe infections in immunocompromised individuals, such as infections at burn sites or in the lungs of patients with immune deficiencies, potentially threatening patient lives [7,8]. *P. syringae* pv. *tomato* DC3000 infects the economically significant tomato crop, resulting in symptoms such as leaf spot disease and substantial economic losses [9,10].

The ferric uptake regulator (Fur) has been identified in Gram-negative bacteria as a global regulator of iron homeostasis, modulating iron uptake (such as siderophores), utilization, and storage processes [11]. Moreover, Fur functions as a global virulence regulator in numerous pathogens, particularly in clinically significant human strains [12,13]. Fur activates the expression of virulence factors such as exotoxin, endoprotease, and extracellular proteases, which are directly implicated in the pathogenicity of *P. aeruginosa* [14]. During *P. syringae* pv. *tabaci* 6605 infection of tobacco, Fur is essential for virulence development [15]. In *Salmonella typhimurium*, *clpV*, the core gene of the type VI secretion system (T6SS) that is closely associated with virulence factor secretion, is significantly upregulated during infection. In summary, the regulation of virulence by Fur in pathogenic bacteria is not contingent upon its control of iron acquisition [16].

*P. donghuensis* HYS, isolated from East Lake in Wuhan, China, exhibits significantly higher iron acquisition capability compared to commonly encountered *Pseudomonas* species, producing 3–4 times more siderophores under iron-restricted conditions [17,18]. The high-yield siderophore was identified as 7-Hydroxytropolone (7-HT) [19]. Moreover, HYS exhibits intense toxicity toward the model organism *Caenorhabditis elegans* [20,21], though the mechanisms underlying its strong pathogenicity and key virulence factors remain poorly understood. *C. elegans* has been widely employed to study host–pathogen interactions due to its small size, short lifespan, simple anatomy, and conserved immune pathways with humans [21].

Although Fur has been well characterized as a global regulator of iron metabolism and virulence in bacteria, existing studies predominantly focus on its regulation of known pathogenic mechanisms. The specific function of Fur in the unknown mechanism of *Pseudomonas* pathogenicity towards *C. elegans*, particularly in the context of HYS’s robust iron acquisition capabilities and pronounced toxicity, remains inadequately explored. In this study, we demonstrate that HYS and PA14 exhibit distinct pathogenic processes, and different pathogenic mechanisms against *C. elegans*. We found that HYS disrupts iron metabolism in nematodes, leading to the hypothesis that Fur may play a significant role in this process. The knockout of *fur* followed by the infection of *C. elegans* revealed that Fur is crucial in the disruption of *C. elegans* iron metabolism by HYS. To further elucidate the regulatory function of Fur in the virulence process of HYS, we utilized bacterial transcriptomics in conjunction with the VFDB to identify the virulence factors regulated by Fur and validate their functions. This study provides novel insights into Fur-regulated virulence factors and advances the understanding of pathogenicity in *Pseudomonas*.

## 2. Materials and Methods

### 2.1. Bacterial Strains, Nematode Strains, and Culture Conditions

Wild-type *C. elegans* Bristol N2 and *Escherichia coli* strain OP50 were obtained from the Caenorhabditis Genetics Center (CGC, University of Minnesota, St. Paul, MN, USA). *E. coli* OP50 is an uracil auxotroph (*Δura*), a genetic deficiency that intrinsically limits its proliferation on Nematode Growth Medium (NGM), thereby preventing overgrowth that could interfere with nematode development. *Pseudomonas donghuensis* strain HYS was isolated from Donghu Lake (30°32′ N, 114°23′ E), a freshwater ecosystem in Wuhan, China, through selective enrichment culture using iron-limited medium. *E. coli* OP50 and pathogenic *P. aeruginosa* PA14 were cultured in LB medium at 37 °C for 12 h. The pathogenic strain HYS, *∆fur*, *∆gspE*, and *∆dddA* were cultured in LB medium at 30 °C for 12 h. Wild-type N2 Bristol strain *C. elegans* was fed with *E. coli* OP50 on NGM plates (6 g NaCl, 5 g peptone, and 1.7% (wt/vol) agar dissolved in deionized water to 1950 mL, sterilized at 115 °C for 30 min, followed by the addition of 2 mL of 1 M CaCl_2_, 2 mL of 1 M MgSO_4_, and 10 mL of phosphate buffer (1 M KH_2_PO_4_, 0.1 M K_2_HPO_4_, and pH = 6.8)) and maintained at 22 °C.

### 2.2. Nematode Lethality Assay

Prior to the lethality assay, wild-type N2 nematodes were transferred onto NGM plates smeared with 30 μL of lysis solution (0.5 M NaOH, 1% NaClO) and incubated at 22 °C for 3 days. To synchronize nematodes, a few lysed adults were then placed on fresh NGM plates smeared with 300 μL OP50 suspension and incubated at 22 °C for 2 h for egg-laying, followed by the removal of adult nematodes. For the lethality assay, 100 adult nematodes were transferred to NGM plates smeared with pathogenic bacteria, incubated at 22 °C, and their mortality was recorded every 24 h, by transferring to fresh pathogen plates as needed. Nematodes were considered dead if they were immobile and unresponsive to physical stimuli, and those without bodies were considered censored.

### 2.3. Construction of Gene-Knockout Strains of P. donghuensis HYS

Suicide plasmid pEX18Gm was utilized for gene knockout through homologous arm recombination, with the primers used for knockout listed in Table S4. The upstream and downstream homologous arms were amplified using specific primers, and the pEX18Gm plasmid was digested with restriction endonucleases. Subsequently, seamless cloning was performed to ligate the two fragments with pEX18Gm, which was then transformed into *E. coli* S17-1 (λpir). Following this, it was conjugated with HYS, and selection was conducted on plates containing gentamicin and chloramphenicol. After isolating the target strain, the aforementioned antibiotics were added to LB medium for overnight culture, followed by a 1:100 transfer into antibiotic-free LB medium for 12 h. Finally, 10% (wt/vol) sucrose LB plates were inoculated, and outer primers were used to verify gene knockout. The reagents and chemicals used included a DNA gel purification kit (Vazyme, Nanjing, China), a plasmid extraction kit (Vazyme, Nanjing, China), a seamless cloning kit (Fanting, Wuhan, China), restriction endonucleases (Thermo Fisher, Waltham, MA, USA), gentamicin at 50 μg/mL concentration, and chloramphenicol at 25 μg/mL concentration.

### 2.4. Pharyngeal Pumping Rate and Defecation Interval Calculation

To observe changes in pharyngeal pumping rates due to pathogenic bacteria, nematodes were synchronized and transferred to pathogen plates. After 24 h, the NGM plates containing nematodes were placed under Leica DM 1000 microscope(4×) (Leica, Wetzlar, Germany) to observe nematodes’ heads, tracking and timing pharyngeal pumping for 30 s. For defecation interval observations, the tail muscle contractions during excretion were timed from the first to the second contraction, defining one defecation interval.

### 2.5. Nematode Gut Colonization Assay

To assess pathogenic bacteria colonization in nematodes, synchronized nematodes were exposed to pathogens at 22 °C for 24 h and paralyzed using 750 μL of 25 mM levamisole solution. The nematodes were centrifuged at 200× *g* for 30 s, resuspended in PBS, washed 3–4 times, then transferred to PBS containing 15 mg/mL chloramphenicol to inhibit other bacteria for 1 h, and finally transferred to 0.3% Triton at room temperature for 4 h to lyse. A 100 μL volume of the suspension was spread on LB agar plates to calculate Colony-Forming Units (CFUs), estimating pathogen colonization per nematode.

### 2.6. Nematode Food Preference and Avoidance Assay

To test nematode preference for pathogens, 50 μL cultures of different pathogens in LB medium were spotted at opposite ends of NGM plates, and synchronized nematodes were placed in the center. Their distribution was recorded every 6 h, calculating the choice index (CI) as (*C. elegans* on pathogen lawn − *C. elegans* on OP50 lawn)/(all *C. elegans* on NGM plate). For avoidance assays, 50 μL of pathogen culture was spotted at the center of NGM plates, with synchronized adults placed at the center, counting nematodes every 2 h to determine avoidance.

### 2.7. RNA Sequencing Sample Collection

A large number of synchronized nematode adults were transferred to pathogen plates, incubated at 22 °C for 24 h, then transferred to 750 μL saline on ice to paralyze them. Three biological replicates were prepared for each pathogen, each containing 1000 adults. Post-collection, nematodes were centrifuged at 200× *g* for 30 s at 4 °C, resuspended in saline, repeating thrice until clear supernatant was obtained, and rapidly stored in liquid nitrogen. For the bacterial samples, HYS, colonizing the intestinal tract of *C. elegans*, was subjected to Dual-RNA sequencing. Approximately 10^10^ bacterial cells of HYS and *Δfur*, grown on NGM plates for 24 h, were collected by rinsing with physiological saline.

### 2.8. RNA Extraction

For RNA extraction, the collected frozen nematodes were ground rapidly from liquid nitrogen and processed using an RNA extraction kit (Vazyme, Nanjing, China). The ground nematodes were resuspended in a lysis buffer, added to a genomic DNA removal column, and centrifuged at 12,000 rpm for 1 min; the filtrate was collected, and RNA was adsorbed, washed twice, and eluted using RNase-free water. For bacteria samples, RNA was extracted using the Trizol method. Following the lysis of bacterial cells, guanidine isothiocyanate in Trizol was employed to denature proteins and inhibit the RNase activity. RNA was then separated from the aqueous phase through chloroform partitioning, and total RNA was precipitated with isopropanol. Subsequently, impurities were removed by ethanol washing, and the final product was dissolved in nuclease-free water. All samples were stored at −80 °C and sent to Majorbio (Shanghai, China) for RNA-seq on the NovaSeq XPlus platform.

### 2.9. RNA Sequencing Data Analysis

The raw data of *C. elegans*, the bacteria, and Dual-RNA sequencing have been uploaded to the NCBI GEO database (accession number: GSE291743). Raw sequencing data were first quality-controlled using FASTX_TOOLKIT (Version 0.0.14), evaluated with FASTAQ (0.19.5), and ribosomal RNA contamination was assessed via the Rfam database (Version14.8). Sequence alignment was performed using STAR (Version 2.7.1a). Gene annotation utilized the NR database (Version 2022.10), Pfam database (Version 35.0), KEGG database (Version 2022.10), eggNOG database (Version 2020.06), Swiss-prot database (Version 2022.10), and GO database (Version 2022.0915). Expression analysis was conducted using Salmon (Version 1.3.0) and DESeq2 (version 1.24.0) and edgeR (version 3.24.0) were employed to compare differentially expressed genes, effectively implementing Benjamini–Hochberg FDR control in genome-wide comparisons. Gene enrichment analysis was performed using the KEGG, GO, and COG databases.

### 2.10. Data Processing

All data are presented as mean ± standard deviation, with each experiment repeated at least thrice. Kaplan–Meier survival analysis was conducted using IBM SPSS Version 23.0 (SPSS Inc., Chicago, IL, USA). Interactions among all groups were compared using the Log-Rank test, followed by post hoc comparisons employing Tukey’s Honestly Significant Difference (Tukey’s HSD). Statistical analysis and plotting were executed using Origin2018 (OriginLab, Northampton, MA, USA), with gene expression volcano plots, heatmaps and KEGG enrichment analysis performed using ggvolcano (https://github.com/BioSenior/ggVolcano, accessed on 26 February 2024), clusterProfiler (3.14.0), and ggplot2 (3.1.0) in R 3.5.2. Three group datasets have been thoroughly reanalyzed using ANOVA with Šidák correction, with datasets violating homogeneity of variance addressed through Welch-corrected ANOVA and Games–Howell post hoc comparisons.

### 2.11. VFDB Prediction

Protein sequences from the virulence factor database (VFDB) (mgc.ac.cn) were downloaded on 16 August 2024, along with Diamond (version 5.0). HYS proteome sequences were aligned, selecting candidates with identity ≥ 40% and *p*-value < 0.05 as potential virulence factors.

## 3. Results

### 3.1. HYS Exhibits Stronger Toxicity Toward C. elegans than PA14

**Figure 1:**
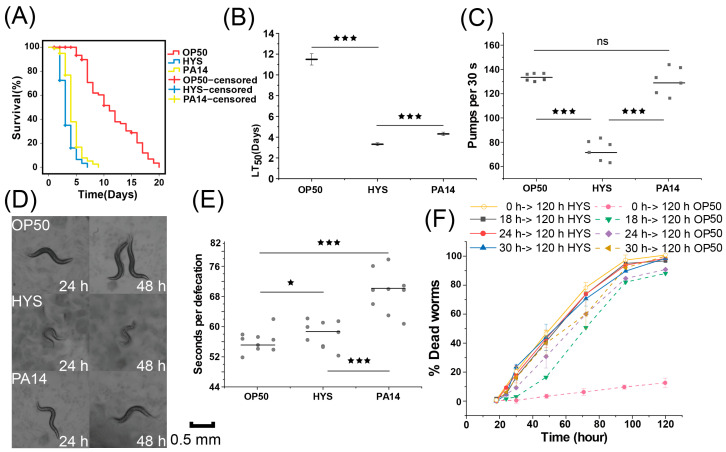
Comparison of the virulence of HYS and PA14 against *Caenorhabditis elegans.* (**A**) The survival curves of *C. elegans* in a slow-killing assay when exposed to OP50, HYS, and PA14. (**B**) The median lethal time (LT_50_) for *C. elegans* exposed to OP50, HYS, and PA14 is presented, with the data indicating the average plus or minus the standard deviation from three independent experiments. *** *p*_adjusted_ < 0.001. (**C**) After a 24 h exposure to HYS and PA14, the pharyngeal pumping frequency over a 30 s interval was recorded for six nematodes, each measured three times for an average value. The gray solid squares represent the mean values obtained from three statistical measurements for each nematode. Error bars represent the mean ± standard deviation. *** *p*_adjusted_ < 0.001, one-way ANOVA followed by Šidák adjustment for multiple comparisons. (**D**) *C. elegans* exposed to OP50, HYS, and PA14 was photographed at 24 and 48 h. (**E**) The defecation timing for 10 nematodes fed on different lawns was recorded after 24 h exposure to HYS and PA14. Each nematode was assessed three times to calculate an average value. The gray solid circles indicate the mean values derived from three statistical assessments for each nematode. Error bars represent the mean ± standard deviation. *** *p*_adjusted_ < 0.001, * *p*_adjusted_ < 0.05, one-way ANOVA followed by Šidák adjustment for multiple comparisons. (**F**) *C. elegans* was exposed to HYS for 18, 24, and 30 h, then transferred to fresh OP50 plates, and two groups of nematodes continuously growing on HYS plates and OP50 plates were used as controls. Mortality was recorded every 24 h until 120 h, with the data expressed as the mean ± standard deviation of three independent experiments.

To thoroughly analyze the characteristics of HYS pathogenicity and explore the underlying mechanisms, a comparative analysis was performed of the chronic lethality of *C. elegans* when fed with the classic human opportunistic pathogen *P. aeruginosa* PA14 and *P. donghuensis* HYS, and *C. elegans* fed with *E. coli* OP50 as negative control (Figure 1A and Figure S1A). The median lethal time (LT_50_) for *C. elegans* fed with HYS was 3.30 ± 0.15 days, compared to 4.42 ± 0.20 days for those fed with PA14 (Figure 1B); at 48 h, the total mortality rate of *C. elegans* fed with HYS surpassed 60%, while those exposed to PA14 only exceeded 20%, indicating a significant difference and demonstrating that HYS exhibits more rapid and intense pathogenicity toward *C. elegans* than PA14.

*C. elegans* exposed to HYS exhibited noticeable sluggish movement within a short period (Figure 1D), with significantly transparent nematodes surface, disorganized internal tissue arrangement, and significant body size reduction. To determine the critical timepoint of HYS pathogenicity, *C. elegans* was transferred to OP50 biofilm after 18, 24, and 30 h of exposure to HYS and PA14, respectively, and mortality rates were recorded. The results (Figure 1F) showed that for *C. elegans* transferred from HYS to OP50 lawns, the mortality rate approached 100% at all timepoints when all *C. elegans* continuously fed with HYS had died. In contrast, for *C. elegans* transferred from PA14 [22], the mortality rate was 95% at 30 h and only 25% at 18 h, indicating that HYS’s pathogenic period occurs before 18 h, much earlier than that of PA14, and the pathogenicity caused by HYS was irreversible.

To investigate whether the rapid pathogenicity of HYS was due to a rapid increase in bacterial load, the pharyngeal pumping frequency and defecation time of *C. elegans* were measured after 24 h of exposure to the pathogens (Figure 1C,E). The pharyngeal pumping frequency of *C. elegans* fed with HYS (71.5 ± 8.26 pumps/30 s) was halved compared to those fed with OP50 (133.5 ± 2.88 pumps/30 s), while the frequency for those fed with PA14 showed no significant change (129.16 ± 2.18 pumps/30 s). The defecation duration of *C. elegans* exposed to PA14 (70.33 ± 7.76 s/event) was also higher than those exposed to HYS (59.33 ± 4.13 s/event). Therefore, with slower ingestion and accelerated defecation, the amount of HYS ingested was much lower than PA14, ruling out a rapid bacterial load increase as the cause of early severe lethality.

Consequently, we conclude that in the chronic lethality assay against *C. elegans*, HYS exhibits stronger toxicity toward *C. elegans* than PA14 and has an irreversible pathogenic process, but not due to rapid bacterial load increase, suggesting that the mechanism of HYS pathogenicity differs from that of PA14.

### 3.2. HYS Infestation Disrupts Iron Metabolism in C. elegans

HYS possesses a more robust iron uptake system [18,23], which may affect the iron metabolism of *C. elegans*. To identify and validate the potential targets sites of HYS attack, transcriptomic sequencing was conducted on *C. elegans* infected by HYS and compared to *C. elegans* fed with OP50. For each transcriptome sample, over 62 million reads were detected, with an average error rate below 0.0248% and Raw Q30 exceeding 95% (Table S1). Replicates clustered well, demonstrating robust biological consistency (Figure S2A,B), ensuring reliability for downstream analysis.

**Figure 2:**
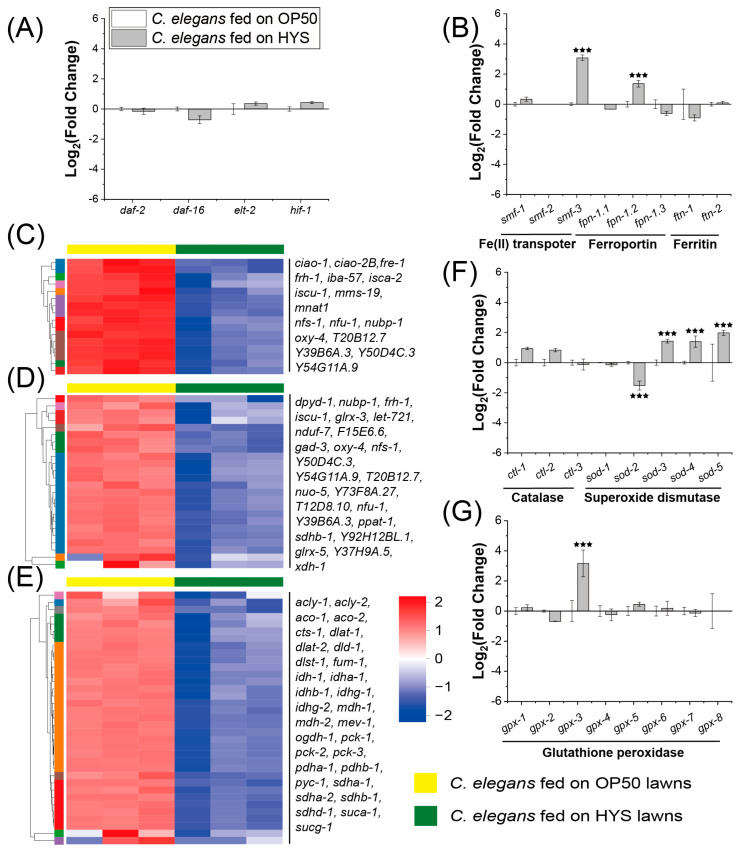
Infection with HYS leads to gene responses related to iron metabolism in *C. elegans*. (**A**,**B**) Expression of iron regulation, iron transport, and iron storage genes is altered in *C. elegans*. White squares denote genes in *C. elegans* fed on OP50; light gray squares indicate genes in *C. elegans* fed on HYS. Expression fold changes are calculated using Log_2_, with *** FDR-adjusted *p* < 0.001 (*p*_adjusted_ < 0.001), was determined through Wald test (DESeq2) and quasi-likelihood F-test (edgeR) with inter-replicate variability shrinkage. (**C**–**E**) Clustered heatmaps showcase expression differences in genes associated with iron–sulfur clusters assembly, iron–sulfur cluster binding, and citrate cycle pathways in *C. elegans*; yellow solid squares indicate gene expression in worms fed on OP50; green solid squares represent gene expression in worms fed on HYS. (**F**,**G**) Expression of catalase, superoxide dismutase, and glutathione peroxidase genes is altered in *C. elegans*. White squares denote genes in *C. elegans* fed on OP50; light gray squares indicate genes in *C. elegans* fed on HYS. Expression fold changes are calculated using Log_2_, with *** FDR-adjusted *p* < 0.001 (*p*_adjusted_ < 0.001), was determined through Wald test (DESeq2) and quasi-likelihood F-test (edgeR) with inter-replicate variability shrinkage.

To investigate whether *C. elegans* infected by HYS exhibits iron metabolic abnormalities, the expression levels of genes involved in iron metabolism regulation, iron transport and storage, and iron utilization between HYS-infected *C. elegans* and those fed with OP50. The expression of iron metabolism-regulatory genes [24,25] (*daf-2*, *daf-16*, *hif-1*, and *elt-2*) showed no significant changes (Figure 2A). In contrast, for downstream genes regulating iron transport (Fe(II) transporter *smf* and ferroportin *fpn*) and storage (ferritin *ftn*) [25], the expression of *smf-3*, and *fpn-1.2* was significantly upregulated, while that of iron storage genes exhibited no substantial changes (Figure 2B). The concurrent upregulation of intracellular iron uptake and export proteins suggests that iron metabolic dysregulation occurred in *C. elegans*. To assess iron utilization, the expression levels of Fe-S clusters assembly and binding proteins were analyzed (Figure 2C,D), which are closely linked to Fe-S clusters abundance, directly impacts iron availability and iron metabolism [26,27]. Fe-S clusters assembly-related genes in HYS-fed *C. elegans* exhibited a downward trend compared to those in OP50-fed *C. elegans*, with 8 out of 16 detected genes showing significant downregulation. Similarly, Fe-S clusters binding genes followed a similar trend, with 9 out of 25 detected genes significantly downregulated in HYS-fed nematodes. These findings indicate that HYS infection severely disrupts the expression of genes involved in Fe-S clusters assembly and binding, consequently impairing iron metabolism in *C. elegans*. iron–sulfur clusters are directly involved in the TCA cycle [28,29], and the intracellular oxidative stress levels indicate the iron metabolism status [30]. To further confirm the disruption of iron metabolism, the downstream pathways affected by iron metabolism were examined, including genes associated with TCA cycle and ROS. Compared to *C. elegans* fed with OP50, the TCA cycle genes in HYS-infected nematodes showed a general decline, with 14 genes being significantly downregulated. The expression of genes related to catalase, superoxide dismutase, and glutathione peroxidase were measured to evaluate the oxidative stress levels. In HYS-infected *C. elegans*, *sod-2* was significantly downregulated, while *sod-3*, *sod-4*, and *sod-5* were significantly upregulated, and *gpx-3* was also significantly upregulated. These two indicators further confirm the dysregulation of iron metabolism in HYS-infected nematodes.

In summary, HYS disrupts the host’s iron metabolic pathways, particularly those related to iron utilization. The disruption of *C. elegans* iron metabolism may stem from HYS’s robust iron uptake capacity or its iron metabolic processes. The Fur’s regulatory role in the iron metabolism of bacteria is likely a critical factor contributing to the HYS-induced disruption of *C. elegans* iron metabolism.

### 3.3. Fur Critically Influences HYS Virulence

**Figure 3:**
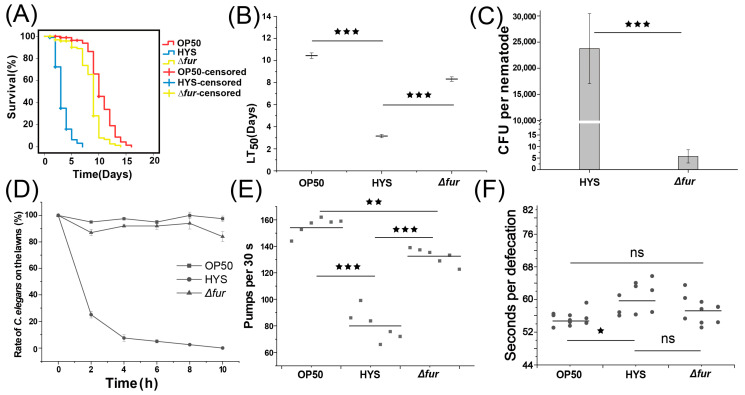
Fur contributes greatly to the virulence of the HYS. (**A**) The survival curves for *C. elegans* subjected to slow-killing assays on HYS and *∆fur* lawns. (**B**) The LT_50_ of *C. elegans* exposed to HYS and *∆fur*, with data shown as the mean ± standard deviation from three independent experiments. *** *p*_adjusted_ < 0.001. (**C**) *C. elegans* exposed for 24 h to HYS and *∆fur*, and colony-forming units (CFUs) were counted. *** *p*_adjusted_ < 0.001. (**D**) *C. elegans* were exposed to OP50, HYS, and *∆fur* lawns, and the number of nematodes on the three types of moss was counted every two hours. (**E**) Following a 24 h exposure to HYS and *∆fur*, the pharyngeal pumping frequency of *C. elegans* was assessed over a 30 s interval. This was performed for six nematodes, with each nematode’s frequency being recorded three times to determine the mean value. The gray solid squares represent the mean values obtained from three statistical measurements for each nematode. Error bars represent the mean ± standard deviation. ** *p*_adjusted_ < 0.01, *** *p*_adjusted_ < 0.001, one-way ANOVA followed by Šidák adjustment for multiple comparisons. (**F**) The defecation timing for ten nematodes fed on different lawns for 24 h was recorded after exposure to HYS and *∆fur*, with each nematode’s timing measured three times to calculate an average value. The gray solid circles indicate the mean values derived from three statistical assessments for each nematode. All data, representing the mean ± standard deviation from three independent experiments, * *p*_adjusted_ < 0.05, while ns denotes no significant difference. Welch’s corrected ANOVA and Games-Howell Post Hoc test for multiple comparisons.

To assess whether the regulatory role of Fur on iron metabolism contributes to HYS-induced pathogenesis in *C. elegans*, chronic lethality experiments were conducted by exposing *C. elegans* to HYS and *Δfur* mutants (Figure 3A,B and Figure S1C). The deletion of *fur* extended the LT_50_ from 3.24 ± 0.15 days to 8.3 ± 0.20 days, significantly attenuating HYS virulence and negating its rapid pathogenicity against *C. elegans*.

Previous research has shown that HYS colonize *C. elegans* extensively within 24 h [21]. To determine whether Fur deletion attenuates HYS virulence by impairing colonization, an intestinal colonization assay was performed (Figure 3C). While wild-type HYS effectively colonized *C. elegans*, *Δfur* failed to establish colonization. Reduction in colonization could result from several factors. First, we examined whether Fur deletion impacted strain palatability or caused avoidance behavior in *C. elegans*. Food avoidance and palatability assays were conducted (Figure 3E,F). After 10 h on NGM plates coated with a single bacterial lawn, *C. elegans* avoided HYS moss entirely, while remaining on *∆fur* and OP50 lawns with >80% occupancy. Additional assays quantified strain palatability by comparing *C. elegans* preference between two bacterial lawns placed at opposite ends of the plate (Figure S1D–F). Over time, nematodes migrated away from HYS lawns (Competition Index [CI] = −0.73 ± 0.03) compared to the neutral response for *∆fur* lawns (CI = −0.02 ± 0.01) (Figure S1B). These findings confirm that HYS was significantly less palatable than *∆fur*, eliminating the possibility that colonization failure resulted from *C. elegans* avoiding the ingestion of *∆fur*. Other factors, including feeding rate and excretion, were also evaluated for their role in colonization failure. The pharyngeal pumping rates of *C. elegans* fed with each strain were measured.

Compared to OP50-fed nematodes (155.611 ± 5.88 events/30 s), feeding on *∆fur* resulted in no significant difference (132.778 ± 5.51 events/30 s). However, HYS-fed nematodes exhibited a significantly reduced pumping rate (80.39 ± 10.71 events/30 s) compared with *∆fur*-fed *C. elegans*. Excretion rates showed no significant difference between HYS-fed (59.86 ± 3.77 events/30 s) and *∆fur*-fed *C. elegans* (57.42 ± 3.21 events/30 s).

Thus, the colonization failure of *∆fur* was not due to ingestion avoidance or reduced feeding but rather a different mechanism. These results suggest that Fur plays a pivotal regulatory role in the acute virulence of HYS against *C. elegans*.

### 3.4. Fur Is Critical in HYS-Mediated Disruption of Iron Metabolism in C. elegans

To investigate whether Fur contributes to the regulation of HYS virulence factors that disrupt iron metabolism in *C. elegans*, transcriptomic analysis was performed on *Δfur*-infested nematodes. Biological replicates clustered well (Figure S2C,D), indicating reliable data for downstream analysis.

**Figure 4:**
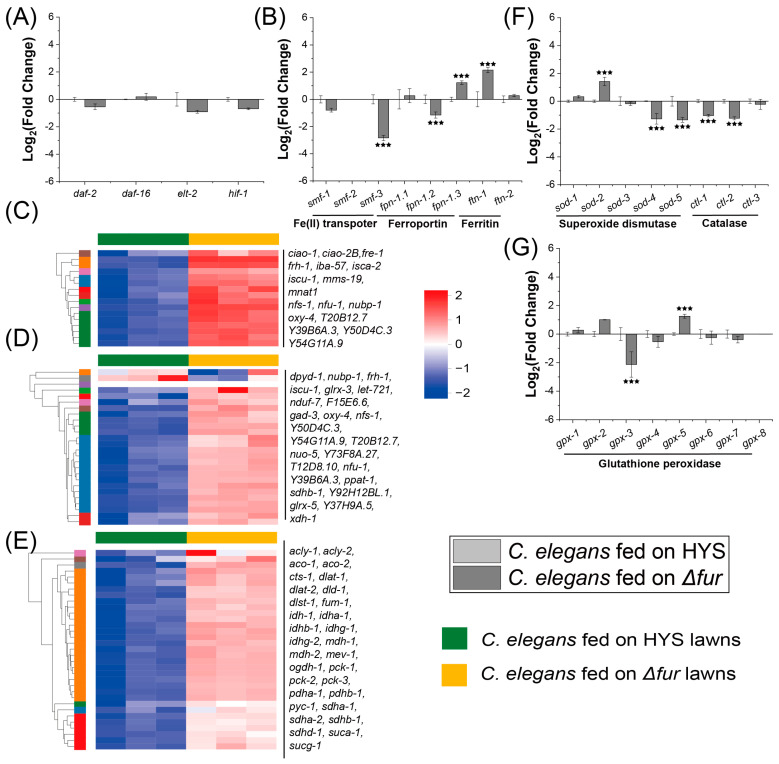
Fur plays a critical role in disrupting iron metabolism in *C. elegans* by HYS. (**A**,**B**) Expression of iron regulation, iron transport, and iron storage genes is altered in *C. elegans*. Light gray squares indicate genes in *C. elegans* fed on HYS, and dark gray squares represent genes in *C. elegans* fed on *∆fur*. Expression fold changes are calculated using Log_2_, with *** FDR-adjusted *p* < 0.001 (*p*_adjusted_ < 0.001), was determined through Wald test (DESeq2) and quasi-likelihood F-test (edgeR) with inter-replicate variability shrinkage; (**C**–**E**) clustered heatmaps showcase expression differences in genes associated with the iron–sulfur cluster assembly, iron–sulfur clusters binding, and citrate cycle pathways in *C. elegans*; green solid squares represent gene expression in worms fed on HYS, and light orange solid squares denote gene expression in worms fed on *∆fur*. (**F**,**G**) Expression of catalase, superoxide dismutase, and glutathione peroxidase genes is altered in *C. elegans*. Light gray squares indicate genes in *C. elegans* fed on HYS. Expression fold changes are calculated using Log_2_, with *** FDR-adjusted *p* < 0.001 (*p*_adjusted_ < 0.001), was determined through Wald test (DESeq2) and quasi-likelihood F-test (edgeR) with inter-replicate variability shrinkage.

Comparing *C. elegans* infected with *Δfur* and those fed with HYS (Figure 4A), the expression levels of iron regulatory genes (*daf-2/daf-16* [31], *hif-1*, and *elt-2* [32]) showed no significant differences. For iron transport and storage genes, *smf-3*, and *fpn-1.2* were significantly downregulated in nematodes cultured on *Δfur* lawns compared to HYS-infected nematodes (Figure 4B). Relative to nematodes cultured on OP50 lawns, *smf* and *fpn* expression levels in *Δfur*-infected nematodes were restored to normal levels. For Fe-S clusters assembly and binding proteins, as well as the TCA cycle-related genes (Figure 4C,E), the expression of most genes in *Δfur*-infected nematodes was significantly higher compared to HYS-infected nematodes, returning to normal levels. The expression levels of superoxide dismutase genes *sod-2*, *sod-4*, and *sod-5* were significantly downregulated, restoring levels to those observed in OP50-fed nematodes (Figure 4F). Similarly, *gpx-3* expression was significantly reduced compared to HYS-fed nematodes (Figure 4G). However, *ftn-1* was significantly upregulated (Figure 4B), *sod-3* and *gpx-5* were significantly upregulated in *Δfur*-infected nematodes compared to OP50-fed nematodes (Figure 4F,G), indicating that *Δfur* can also induce some degree of oxidative stress.

In summary, although the absence of Fur induces some oxidative stress, the disruption of key pathways involved in iron metabolism, such as iron absorption, transport, and utilization, has been mitigated. Therefore, we conclude that the Fur protein plays a crucial regulatory role in the iron metabolism of *C. elegans* affected by HYS.

### 3.5. Screening of Fur-Regulated Virulence Factors in HYS

Virulence factors serve as direct “weapons” that enable pathogenic bacteria to establish infection [22], and investigating these factors is essential for elucidating bacterial pathogenic mechanisms [33]. To further investigate the regulatory role of Fur in the virulence exerted by HYS, we aimed to identify the virulence factors regulated by Fur during HYS infection, and we attempted to screen for these factors by analyzing the transcriptomes of pathogenic bacteria under various conditions. For the pathogenic bacterial samples, we utilized HYS colonizing the intestines of *C. elegans*, as well as HYS and *Δfur* grown on NGM plates, resulting in the detection of over 21 million reads (Table S1), with an average raw error rate below 0.0254% and raw Q30 values exceeding 90%. Biological replicates for each sample were consistent, with similar biological replicates clustering well (Figure S2A,B); thus, these data were suitable for downstream analysis.

**Figure 5:**
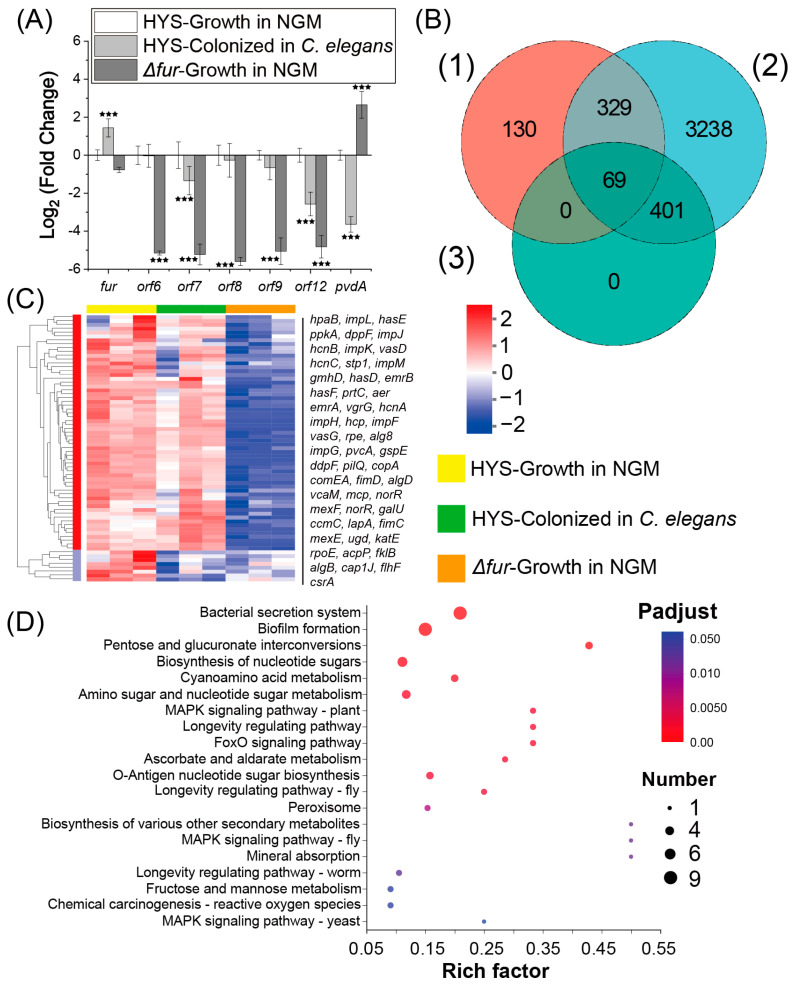
Screening of virulence factors that function in nematodes in Fur-regulated HYS strains. (**A**) Bar chart showing fold changes in expression of *fur*, *orf9-6*, *orf12*, and *pvdA* within HYS colonizing nematodes and HYS and *∆fur* grown on NGM, where light gray squares indicate HYS within nematodes, white squares represent HYS grown on NGM, and dark gray squares denote *∆fur* grown on NGM; expression fold changes are processed with Log_2_, with *** FDR-adjusted *p* < 0.001 (*p*_adjusted_ < 0.001), was determined through Wald test (DESeq2) and quasi-likelihood F-test (edgeR) with inter-replicate variability shrinkage. (**B**) Venn diagram of the screening of virulence factors regulated by Fur. The orange circles represent the set of genes that were down-regulated by *∆fur* cultured on NGM plates compared to HYS cultured on NGM plates. Blue circles represent the set of genes whose transcript levels were not significantly reduced between HYS colonized in nematodes and HYS grown on NGM plates. The green circles represent the 469 virulence factor gene sets predicted by VFDB in the HYS genome. (1)–(3) represent the three screening criteria mentioned in Result 3.5. (**C**) A clustered heatmap of expression differences for genes related to 69 Fur-controlled within HYS colonizing nematodes and HYS and *∆fur* grown on NGM, where light yellow solid squares indicate gene expression in HYS grown on NGM, laurel-green solid squares represent gene expression HYS within nematodes, and orange solid squares denote gene expression in *∆fur* grown on NGM. (**D**) Bubble plot of KEGG enrichment analysis of virulence factors that may play a role in the virulence of nematodes in 69 Fur-controlled HYS screened.

The expression level of *fur* was significantly upregulated when colonizing the nematodes. Furthermore, siderophores may disrupt host iron metabolism through iron piracy [9,10,34]. To determine whether iron carriers exert virulence during HYS infection, we evaluated the expression levels of synthesis genes of pyoverdine (*pvdA*) and 7-HT (*orf9-6*, *orf12*) in the nematodes (Figure 5A). The results indicated that the expression levels of both iron carriers were significantly downregulated, with the 7-HT synthesis genes showing even more pronounced downregulation following Fur deletion (TPM nearly 0), while *pvdA* was significantly upregulated. This finding contradicts the notion that HYS disrupts iron metabolism in the nematodes, suggesting that neither 7-HT nor pyoverdine participates in the disruption of iron metabolism in *C. elegans*. To further evaluate and identify the virulence factors regulated by Fur during pathogenesis, in order to investigate the critical regulatory role of Fur in the virulence of HYS, the following conditions were employed to screen for virulence factors regulated by Fur, based on the extensive colonization of *C. elegans* by HYS and the positive regulatory influence of Fur on these factors.

(1) Genes not significantly downregulated in HYS colonized in *C. elegans* compared to HYS grown on NGM plates (4037 genes);

(2) Genes significantly downregulated in *Δfur* grown on NGM plates compared to wild-type HYS on NGM plates (528 genes);

(3) Genes matching the VFDB in the HYS genome (469 genes).

Intersecting these datasets yielded 69 target genes (Table S3) (Figure 5C). Expression analysis revealed nine virulence factors significantly upregulated in response to nematode nutritional immunity (Table 1), with many maintaining expression comparable to NGM-grown HYS. KEGG enrichment analysis revealed involvement in pathways such as the bacterial secretion system, biofilm formation, and type II (T2SS) and type VI (T6SS) secretion systems (Figure 5D). Additional matches included pathways related to nucleotide sugar metabolism and oxidative stress mechanisms (peroxisome and chemical carcinogenesis—reactive oxygen species).

In summary, *fur* was significantly upregulated during the infection process, further confirming that Fur plays a crucial regulatory role in the virulence of HYS. However, siderophores, which primarily disrupt host iron metabolism in other pathogenic bacteria-host interaction models, do not function as virulence factors in the interaction between HYS and *C. elegans*. Therefore, the disruption of host iron metabolism by HYS is a process that is independent of siderophores. Moreover, during the regulation of HYS virulence in the context of the nematode, Fur predominantly modulates virulence factors that are more closely associated with biofilm formation and secretion systems.

### 3.6. Validation of Fur-Regulated Virulence Factors

**Figure 6:**
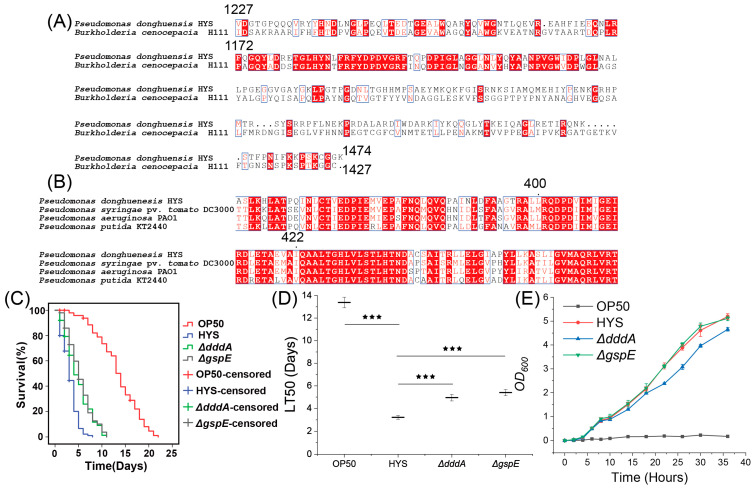
Both DddA and GspE contribute to the virulence of HYS against *C. elegans*. (**A**,**B**) Multiple sequence alignment of DddA and GspE with conserved domains. Red squares indicate regions with identical amino acid sequences, while white squares represent regions with amino acid sequence identity exceeding the threshold (70%). (**C**) Survival curves of *C. elegans* subjected to slow-killing assays on lawns of HYS, *∆dddA*, and *∆gspE*. (**D**) LT_50_ values of C. elegans exposed to OP50, HYS, *∆dddA*, and *∆gspE*, presented as mean ± standard deviation from three independent experiments. *** *p*_adjusted_ < 0.001. (**E**) Growth curves of OP50, HYS, *∆dddA*, and *∆gspE* on NGM plates, with data shown as the mean ± standard deviation from three independent experiments.

To validate the roles of the virulence factors identified through bioinformatics analysis and the aforementioned conditions that are truly capable of executing the functions of virulence factors in HYS. we focused on DddA, whose virulence function remains unstudied, and the structural protein GspE of T2SS, previously unexplored by our research group, performing gene knockouts to assess their attenuated virulence effects. Bioinformatic methods confirmed their annotation accuracy, with multiple sequence alignments identifying conserved domains for DddA and GspE (Figure 6A,B). DddA, a double-stranded DNA deaminase toxin A, was first characterized in the *Burkholderia cenocepacia* strain H111 [35]. Comparisons revealed 39.8% sequence identity and significant similarities in the C-terminal activation region between DddA expressed by HYS and H111 (Figure 6A), confirming the homologous functionality of HYS *dddA*. GspE, a general secretion pathway protein, is integral to bacterial survival and adaptability as an essential component of T2SS [36,37]. Comparisons of HYS-expressed GspE with pathogenic *P. syringae*, *P. aeruginosa*, and *P. putida* revealed conserved structural domains spanning amino acid positions 400–422, affirming its homology to other T2SS GspE proteins. Gene-deletion mutants *∆dddA* and *∆gspE* were constructed, and chronic lethality assays were performed by feeding these strains to *C. elegans* (Figure 6C,D). Nematodes fed OP50 exhibited an LT50 of 13.379 ± 0.466 days, whereas HYS reduced this to 3.221 ± 0.162 days. In contrast, the deletion of *dddA* and *gspE* significantly attenuated HYS virulence, extending LT50 to 4.953 ± 0.29 and 5.399 ± 0.272 days, respectively. These results confirm that DddA and GspE contribute to HYS pathogenicity during *C. elegans* colonization and are regulated by Fur, highlighting their roles as virulence factors.

## 4. Discussion

### 4.1. HYS Exhibits Different Molecular Mechanism Underlying Its Enhanced and Irreversible Pathogenicity

The chronic lethality model of pathogenic bacteria in *C. elegans* serves as a vital approach to understanding the interactions between pathogens and hosts in the context of resource competition. This study analyzed the pathogenic processes of *C. elegans* infected by HYS and PA14, revealing that HYS exhibits more pronounced and irreversible pathogenic outcomes compared to PA14. Pathogenicity assays demonstrated that HYS caused irreversible damage to *C. elegans* within 18 h, whereas PA14 required at least 30 h to induce comparable irreversible harm [22]. Other reported chronic lethality models show similar patterns; for instance, *P. aeruginosa* CF18 fails to cause mortality within the first 40 h of infection [38]. Similarly, *Vibrio cholerae*, a classical human pathogen, inflicts mortality on *C. elegans* only after 3–4 days of contact [39]. By comparing HYS to these pathogens, we observed that, while many species require prolonged infection (over 30 h) to cause nematode death, HYS rapidly induces irreversible mortality within 18 h, highlighting its unique pathogenic dynamics. This suggests that HYS may rapidly produce substantial amounts of potent virulence factors to cause nematode damage. This indicates that the pathogenic mechanism of HYS may differ from that of *P. aeruginosa* PA14 and other pathogens.

### 4.2. HYS Infection Disrupts Iron Metabolism of C. elegans

The potential pathways of *C. elegans* targeted by HYS were analyzed to elucidate the underlying molecular mechanisms of HYS’s strong pathogenicity against *C. elegans*. Given HYS’s superior iron uptake capacity [17,18], we focused on the expression of iron metabolism-related genes in *C. elegans* and found that HYS infection disrupts host iron metabolism pathways.

HYS infection caused the dysregulation of iron absorption (*smf-3*) and iron export (*fpn-1.2*) genes, along with the significant suppression of genes related to iron–sulfur clusters assembly and binding protein synthesis, this indicates that HYS infection severely disrupts the iron metabolism process in *C. elegans*. In *C. elegans* cells, pathways regulating iron metabolism, transport, storage, and utilization function as an integrated system, with gene expression normally unified by iron concentration. *Smf-1* and *Smf-3* localize to the intestinal cells of nematodes and uptake iron and manganese ions [40]. The *fpn* gene is responsible for iron export [41]. If both are simultaneously upregulated abnormally, it may lead to an imbalance in the “import-export” dual channel of intracellular iron ions, resulting in a paradox of iron metabolism characterized by “ineffective cycling”, indicating a disruption in iron metabolism. Currently, there are no reported studies of instances where both the import and export channels are abnormally upregulated. iron–sulfur clusters are vital cofactors in cellular metabolic pathways (such as respiration, the TCA cycle, energy metabolism, and DNA repair), and represent an indispensable form of intracellular iron utilization [42,43]. Furthermore, studies utilizing BT-549 cell lines have revealed that deficiencies in iron–sulfur cluster synthesis can severely impact mitochondrial energy metabolism and promote iron absorption by activating the expression of iron regulatory proteins (IRP), leading to iron metabolism imbalance and oxidative stress, thus disrupting cellular iron metabolism [44]. In *C. elegans*, the knockdown of CDGSH Iron Sulfur Domain (CISD) protein, an iron–sulfur clusters binding protein primarily localized to the mitochondrial membrane, results in an additional increase in intracellular iron concentration, disrupting iron metabolism in *C. elegans* and leading to its neuronal degeneration, and increased levels of proteins related to Parkinson’s and Huntington’s diseases, thus significantly affecting the lifespan of *C. elegans* [45]. Therefore, the abnormal upregulation of iron absorption and iron efflux, along with the significant inhibition of iron–sulfur clusters synthesis and the responses of the TCA cycle and ROS, indicates that HYS infection leads to a significant disruption of iron metabolism in *C. elegans*. This report represents the first documentation of pathogen-induced suppression of iron–sulfur clusters synthesis.

### 4.3. Fur Plays Critical Positive Regulatory Role in HYS-Mediated Disruption of C. elegans Iron Metabolism

Our findings revealed that Fur plays an essential role in HYS-induced irreversible severe pathogenicity and acts as a critical positive regulator in HYS’s disruption of *C. elegans* iron metabolism. We observed that *Δfur* significantly attenuated HYS’s pathogenicity toward *C. elegans*, and the irreversible pathogenic features were eliminated. Furthermore, Fur was essential for HYS colonization in the host. In the mouse infection model, the colonization efficiency of the *Helicobacter pylori fur* mutant was lower than that of the wild type strain. In a competition assay using *Mongolian gerbils*, the *fur* mutant of *H. pylori* was readily outcompeted by the wild-type strain [46] in accordance with the classical virulence regulatory function of Fur. Moreover, infection with *Δfur* restored *C. elegans* iron transport and iron utilization to near-normal levels, indicating that Fur positively regulates virulence factors secreted by HYS that disrupt *C. elegans* iron metabolism.

Transcriptomic sequencing was performed under various conditions to identify the Fur-regulated virulence factors. Although Fur regulates the synthesis of 7-HT and pyoverdine in HYS, these two siderophores were not involved in the pathogenic process and the disruption of iron metabolism identify the Fur-regulated virulence factors. During lung infections caused by *P. aeruginosa*, pyoverdine and pyochelin were secreted to capture host iron [47,48,49], and Fur facilitates infection by regulating the synthesis of the siderophores, enabling iron acquisition at the infection site [14]. In the infection of tobacco by *P. syringae* pv. *tabaci* 6605, Fur-regulated pyoverdine is essential for virulence development [15]. Similarly, *Salmonella* acquires host intestinal iron through the secretion of enterochalin [50]. Unlike these pathogens that employ siderophore-based strategies to disrupt host iron metabolism [49,50], HYS disrupts host iron metabolism in a non-siderophore-dependent manner. The VFDB was then utilized to screen Fur-regulated virulence factors to further elucidate the regulatory function of Fur [51]. We identified 69 virulence factors, primarily alkaline proteases [52] and proteins associated with T2SS [53]. Among these 69 virulence factors, none have been reported to have functions that disrupt iron metabolism. This suggests that firstly, the functions of these 69 proteins may not yet be fully known, and secondly, it is possible that virulence factors that disrupt iron metabolism have not been included in the VFDB, or that the disruption of host iron metabolism is not the result of a single virulence factor, but rather a consequence of the combined actions of multiple virulence factors. These implications provide direction and justification for subsequent research. DddA and the key T2SS gene GspE were knocked out, and through chronic lethality experiments, we confirmed that GspE and DddA, under the positive regulation of Fur, contribute to HYS’s virulence, particularly the conserved ATPase GspE essential for T2SS assembly.

Future research goals include overcoming the technical barriers for measuring the intracellular iron levels in nematodes during pathogen-nematode interactions, as well as investigating the quantitative levels of host iron–sulfur clusters and associated proteins.

**Figure 7:**
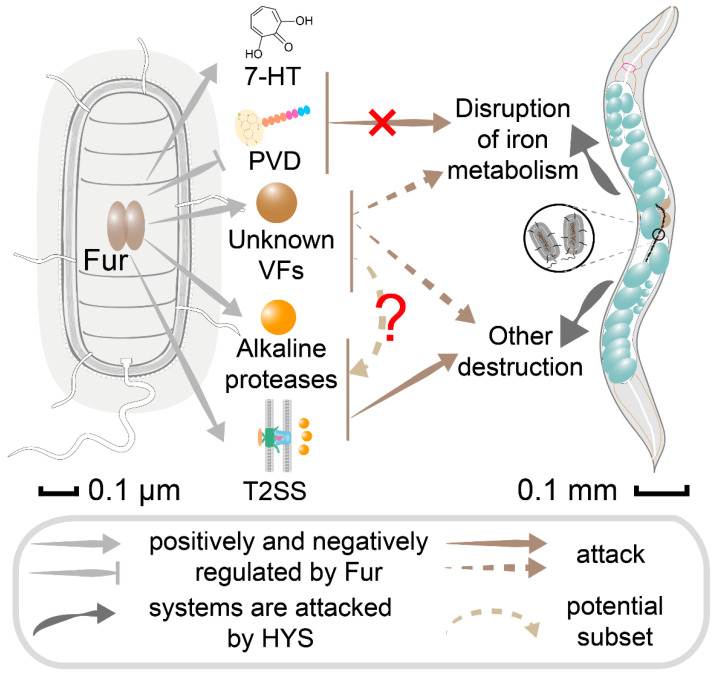
Schematic diagram of HYS interactions with *C. elegans* through Fur-regulated virulence factors. Light gray arrows and T-shaped arrows indicate virulence factors that are positively and negatively regulated by Fur, respectively. Solid brown arrows and dashed brown arrows indicates which host system was targeted by virulence factor. Dark gray arrows indicate systems where host was attacked. The light brown arced dashed arrows and the red question mark represent it is currently undetermined whether the unknown VFs represent a potential subset of the 69 identified virulence factors. PVD-pyoverdine; VFs-virulence factors.

In summary, this study identifies that HYS disrupts the iron metabolism pathway in *C. elegans*, with Fur significantly contributing, and seeks to identify the virulence factors regulated by Fur; it is unclear which factors are directly linked to the disruption of iron metabolism. Expanding on that, this study shows that HYS exhibits significantly stronger and irreversible pathogenicity against *C. elegans* compared to *P. aeruginosa* PA14. HYS infection disrupts nematode iron metabolism (Figure 7), with Fur playing a key positive regulatory role. Siderophores, 7-HT, and pyoverdine do not participate in disrupting the iron metabolism of *C. elegans*; the virulence factors directly regulated by Fur that may associate with the iron homeostasis of *C. elegans* still need to be further explored. To further delineate the regulatory function of Fur, a series of virulence factors were screened, and the positive regulation of virulence factors relevant to HYS virulence by Fur was validated. Furthermore, to the best of our knowledge, this is the first report on pathogenic bacterial infection inhibiting host iron–sulfur clusters synthesis and the first report that *DddA* can function as a virulence factor of the pathogen. This study demonstrates that HYS disrupts *C. elegans* iron metabolism through a Fur-regulated, siderophore-independent pathogenic process, enriching the understanding of the pathogenic mechanisms of *Pseudomonas* and other Gram-negative bacteria and providing new perspectives for subsequent pathogenic research.

## Figures and Tables

**Table 1:** Fur-regulated virulence factors significantly upregulated during colonization in *C. elegans*.

Gene ID	Gene Name	Fold Change	Gene Description
UW3_RS0123895	*hasE*	4.676	Alkaline protease secretion protein
UW3_RS0123890	*hasF*	4.507	Alkaline protease secretion protein
UW3_RS0115515	*dppF*	3.647	Di/tripeptide transport ATP-binding protein
UW3_RS0104960	*gspE*	2.924	General secretion pathway protein E
UW3_RS0105380	*dddA*	2.907	Double-stranded DNA deaminase toxin A
UW3_RS0117820	*comEA*	2.666	Uncharacterized protein
UW3_RS0116245	*ddpF*	2.583	Probable peptide ABC transporter ATP-binding protein
UW3_RS27610	*hasD*	2.094	Alkaline protease secretion ATP-binding protein
UW3_RS0105455	*impL*	2.043	Type VI secretion system component

## Data Availability

The data presented in this study are openly available in https://www.ncbi.nlm.nih.gov/geo (accessed on 13 March 2025) [GSE291743].

## Supplementary Materials

The following supporting information can be downloaded at: https://www.mdpi.com/article/10.3390/microorganisms13051081/s1, Figure S1: Growth curves of pathogens and selective avoidance of different strains by *C. elegans*; Figure S2: Biological duplications between individual samples in the transcriptome and the correlation situation; Table S1: Table of quality control statistics for transcriptome data of *Caenorhabditis elegans* cultured under different conditions; Table S2: Table of quality control statistics for transcriptome data of pathogens under different conditions; Table S3: Table of no significantly down-regulated genes found in HYS colonized in *C. elegans*; Table S4: Table of primers used for gene knockouts.

## References

[1] Hood M.I., Skaar E.P. (2012). Nutritional Immunity: Transition Metals at the Pathogen-Host Interface. Nat. Rev. Microbiol..

[2] Yiannikourides A., Latunde-Dada G.O. (2019). A Short Review of Iron Metabolism and Pathophysiology of Iron Disorders. Medicines.

[3] Van Dingenen J. (2021). Harder, Better, Faster, Stronger: Iron Strengthens Pathogenic Bacteria Too!. Plant Cell.

[4] Wang B., Lin Y.-C., Vasquez-Rifo A., Jo J., Price-Whelan A., McDonald S.T., Brown L.M., Sieben C., Dietrich L.E.P. (2021). *Pseudomonas aeruginosa* PA14 Produces R-Bodies, Extendable Protein Polymers with Roles in Host Colonization and Virulence. Nat. Commun..

[5] Mahboub H.H., Yousefi M., Abdelgawad H.A., Abdelwarith A.A., Younis E.M., Sakr E., Khamis T., Ismail S.H., Abdel Rahman A.N. (2025). Expression Profiling of Antimicrobial Peptides and Immune-Related Genes in Nile Tilapia Following *Pseudomonas putida* Infection and Nano-Titanium Dioxide Gel Exposure. Fish Shellfish Immunol..

[6] Bashir A., Tian T., Yu X., Meng C., Ali M., Li L. (2020). Pyoverdine-Mediated Killing of *Caenorhabditis elegans* by *Pseudomonas syringae* MB03 and the Role of Iron in Its Pathogenicity. Int. J. Mol. Sci..

[7] Lyczak J.B., Cannon C.L., Pier G.B. (2000). Establishment of *Pseudomonas aeruginosa* Infection: Lessons from a Versatile Opportunist. Microbes Infect..

[8] Jurado-Martín I., Sainz-Mejías M., McClean S. (2021). *Pseudomonas aruginosa*: An Audacious Pathogen with an Adaptable Arsenal of Virulence Factors. Int. J. Mol. Sci..

[9] Xin X.-F., He S.Y. (2013). *Pseudomonas syringae* Pv. Tomato DC3000: A Model Pathogen for Probing Disease Susceptibility and Hormone Signaling in Plants. Annu. Rev. Phytopathol..

[10] Jones A.M., Wildermuth M.C. (2011). The Phytopathogen *Pseudomonas syringae* Pv. Tomato DC3000 Has Three High-Affinity Iron-Scavenging Systems Functional under Iron Limitation Conditions but Dispensable for Pathogenesis. J. Bacteriol..

[11] Ratledge C., Dover L.G. (2000). Iron Metabolism in Pathogenic Bacteria. Annu. Rev. Microbiol..

[12] Fillat M.F. (2014). The Fur (Ferric Uptake Regulator) Superfamily: Diversity and Versatility of Key Transcriptional Regulators. Arch. Biochem. Biophys..

[13] Carpenter B.M., Whitmire J.M., Merrell D.S. (2009). This Is Not Your Mother’s Repressor: The Complex Role of Fur in Pathogenesis. Infect. Immun..

[14] Reinhart A.A., Oglesby-Sherrouse A.G. (2016). Regulation of *Pseudomonas aeruginosa* Virulence by Distinct Iron Sources. Genes.

[15] Taguchi F., Suzuki T., Inagaki Y., Toyoda K., Shiraishi T., Ichinose Y. (2010). The Siderophore Pyoverdine of *Pseudomonas syringae* Pv. Tabaci 6605 Is an Intrinsic Virulence Factor in Host Tobacco Infection. J. Bacteriol..

[16] Andrews S.C., Robinson A.K., Rodríguez-Quiñones F. (2003). Bacterial Iron Homeostasis. FEMS Microbiol. Rev..

[17] Gao J., Xie G., Peng F., Xie Z. (2015). *Pseudomonas donghuensis* Sp. Nov., Exhibiting High-Yields of Siderophore. Antonie Van Leeuwenhoek.

[18] Yu X., Chen M., Jiang Z., Hu Y., Xie Z. (2014). The Two-Component Regulators GacS and GacA Positively Regulate a Nonfluorescent Siderophore through the Gac/Rsm Signaling Cascade in High-Siderophore-Yielding *Pseudomonas* sp. strain HYS. J. Bacteriol..

[19] Jiang Z., Chen M., Yu X., Xie Z. (2016). 7-Hydroxytropolone Produced and Utilized as an Iron-Scavenger by *Pseudomonas donghuensis*. Biometals.

[20] Xie G., Zeng M., You J., Xie Z. (2019). *Pseudomonas donghuensis* HYS Virulence towards *Caenorhabditis elegans* Is Regulated by the Cbr/Crc System. Sci. Rep..

[21] Xiao Y., Wang P., Zhu X., Xie Z. (2021). *Pseudomonas donghuensis* HYS gtrA/B/II Gene Cluster Contributes to Its Pathogenicity toward *Caenorhabditis elegans*. Int. J. Mol. Sci..

[22] Tan M.-W., Mahajan-Miklos S., Ausubel F.M. (1999). Killing of *Caenorhabditis elegans* by *Pseudomonas aeruginosa* Used to Model Mammalian Bacterial Pathogenesis. Proc. Natl. Acad. Sci. USA.

[23] Gui Z., You J., Xie G., Qin Y., Wu T., Xie Z. (2020). *Pseudomonas donghuensis* HYS 7-Hydroxytropolone Contributes to Pathogenicity toward *Caenorhabditis elegans* and Is Influenced by Pantothenic Acid. Biochem. Biophys. Res. Commun..

[24] Romero-Afrima L., Zelmanovich V., Abergel Z., Zuckerman B., Shaked M., Abergel R., Livshits L., Smith Y., Gross E. (2020). Ferritin Is Regulated by a Neuro-Intestinal Axis in the Nematode *Caenorhabditis elegans*. Redox Biol..

[25] Anderson C.P., Leibold E.A. (2014). Mechanisms of Iron Metabolism in *Caenorhabditis elegans*. Front. Pharmacol..

[26] Ast T., Meisel J.D., Patra S., Wang H., Grange R.M.H., Kim S.H., Calvo S.E., Orefice L.L., Nagashima F., Ichinose F. (2019). Hypoxia Rescues Frataxin Loss by Restoring Iron Sulfur Cluster Biogenesis. Cell.

[27] Ye H., Rouault T.A. (2010). Human Iron-Sulfur Cluster Assembly, Cellular Iron Homeostasis, and Disease. Biochemistry.

[28] Zhang S., Xin W., Anderson G.J., Li R., Gao L., Chen S., Zhao J., Liu S. (2022). Double-Edge Sword Roles of Iron in Driving Energy Production versus Instigating Ferroptosis. Cell Death Dis..

[29] Cronin S.J.F., Woolf C.J., Weiss G., Penninger J.M. (2019). The Role of Iron Regulation in Immunometabolism and Immune-Related Disease. Front. Mol. Biosci..

[30] Puntarulo S. (2005). Iron, Oxidative Stress and Human Health. Mol. Asp. Med..

[31] Singh V., Aballay A. (2009). Regulation of DAF-16-Mediated Innate Immunity in *Caenorhabditis elegans*. J. Biol. Chem..

[32] James S.A., Roberts B.R., Hare D.J., de Jonge M.D., Birchall I.E., Jenkins N.L., Cherny R.A., Bush A.I., McColl G. (2015). Direct in Vivo Imaging of Ferrous Iron Dyshomeostasis in Ageing *Caenorhabditis elegans*. Chem. Sci..

[33] Vega L.A., Sanson-Iglesias M., Mukherjee P., Buchan K.D., Morrison G., Hohlt A.E., Flores A.R. (2024). LiaR-Dependent Gene Expression Contributes to Antimicrobial Responses in Group A *Streptococcus*. Antimicrob. Agents Chemother..

[34] Takase H., Nitanai H., Hoshino K., Otani T. (2000). Impact of Siderophore Production on *Pseudomonas aeruginosa* Infections in Immunosuppressed Mice. Infect. Immun..

[35] de Moraes M.H., Hsu F., Huang D., Bosch D.E., Zeng J., Radey M.C., Simon N., Ledvina H.E., Frick J.P., Wiggins P.A. (2021). An Interbacterial DNA Deaminase Toxin Directly Mutagenizes Surviving Target Populations. Elife.

[36] Liu H., Xu G., Guo B., Liu F. (2024). Old Role with New Feature: T2SS ATPase as a Cyclic-Di-GMP Receptor to Regulate Antibiotic Production. Appl. Environ. Microbiol..

[37] Filloux A. (2004). The Underlying Mechanisms of Type II Protein Secretion. Biochim. Biophys. Acta.

[38] Mirza Z., Walhout A.J.M., Ambros V. (2023). A Bacterial Pathogen Induces Developmental Slowing by High Reactive Oxygen Species and Mitochondrial Dysfunction in *Caenorhabditis elegans*. Cell Rep..

[39] Vaitkevicius K., Lindmark B., Ou G., Song T., Toma C., Iwanaga M., Zhu J., Andersson A., Hammarström M.-L., Tuck S. (2006). A *Vibrio cholerae* Protease Needed for Killing of *Caenorhabditis elegans* Has a Role in Protection from Natural Predator Grazing. Proc. Natl. Acad. Sci. USA.

[40] Au C., Benedetto A., Anderson J., Labrousse A., Erikson K., Ewbank J.J., Aschner M. (2009). SMF-1, SMF-2 and SMF-3 DMT1 Orthologues Regulate and Are Regulated Differentially by Manganese Levels in *C. elegans*. PLoS ONE.

[41] Rajan M., Anderson C.P., Rindler P.M., Romney S.J., Ferreira Dos Santos M.C., Gertz J., Leibold E.A. (2019). NHR-14 Loss of Function Couples Intestinal Iron Uptake with Innate Immunity in *C. elegans* through PQM-1 Signaling. Elife.

[42] Shi R., Hou W., Wang Z.-Q., Xu X. (2021). Biogenesis of Iron-Sulfur Clusters and Their Role in DNA Metabolism. Front. Cell Dev. Biol..

[43] Vallières C., Benoit O., Guittet O., Huang M.-E., Lepoivre M., Golinelli-Cohen M.-P., Vernis L. (2024). Iron-Sulfur Protein Odyssey: Exploring Their Cluster Functional Versatility and Challenging Identification. Metallomics.

[44] Terzi E.M., Sviderskiy V.O., Alvarez S.W., Whiten G.C., Possemato R. (2021). Iron-Sulfur Cluster Deficiency Can Be Sensed by IRP2 and Regulates Iron Homeostasis and Sensitivity to Ferroptosis Independent of IRP1 and FBXL5. Sci. Adv..

[45] Ploumi C., Kyriakakis E., Tavernarakis N. (2023). Coupling of Autophagy and the Mitochondrial Intrinsic Apoptosis Pathway Modulates Proteostasis and Ageing in *Caenorhabditis elegans*. Cell Death Dis..

[46] Gancz H., Censini S., Merrell D.S. (2006). Iron and pH Homeostasis Intersect at the Level of Fur Regulation in the Gastric Pathogen *Helicobacter pylori*. Infect. Immun..

[47] Cornelis P., Dingemans J. (2013). *Pseudomonas aeruginosa* Adapts Its Iron Uptake Strategies in Function of the Type of Infections. Front. Cell Infect. Microbiol..

[48] Sánchez-Jiménez A., Llamas M.A., Marcos-Torres F.J. (2023). Transcriptional Regulators Controlling Virulence in *Pseudomonas aeruginosa*. Int. J. Mol. Sci..

[49] Minandri F., Imperi F., Frangipani E., Bonchi C., Visaggio D., Facchini M., Pasquali P., Bragonzi A., Visca P. (2016). Role of Iron Uptake Systems in *Pseudomonas aeruginosa* Virulence and Airway Infection. Infect. Immun..

[50] Spiga L., Fansler R.T., Perera Y.R., Shealy N.G., Munneke M.J., David H.E., Torres T.P., Lemoff A., Ran X., Richardson K.L. (2023). Iron Acquisition by a Commensal Bacterium Modifies Host Nutritional Immunity during *Salmonella* Infection. Cell Host Microbe.

[51] Liu B., Zheng D., Zhou S., Chen L., Yang J. (2022). VFDB 2022: A General Classification Scheme for Bacterial Virulence Factors. Nucleic Acids Res..

[52] Laarman A.J., Bardoel B.W., Ruyken M., Fernie J., Milder F.J., van Strijp J.A.G., Rooijakkers S.H.M. (2012). *Pseudomonas aeruginosa* Alkaline Protease Blocks Complement Activation via the Classical and Lectin Pathways. J. Immunol..

[53] Krekhno Z., Woodward S.E., Serapio-Palacios A., Peña-Díaz J., Moon K.M., Foster L.J., Finlay B.B. (2024). *Citrobacter rodentium* Possesses a Functional Type II Secretion System Necessary for Successful Host Infection. Gut Microbes.

