# Supplementary material for: Ferric Uptake Regulator Contributes to Pseudomonas donghuensis HYS-Induced Iron Metabolic Disruption in Caenorhabditis elegans
Source: Microorganisms. 2025 May 6;13(5):1081. doi: 10.3390/microorganisms13051081 (PMC12114261; doi:10.3390/microorganisms13051081)
Supplement: Supplementary file 1 [file microorganisms-13-01081-s001.zip › supplementary materials of FigureS1&2.pdf]

Supplementary Materials

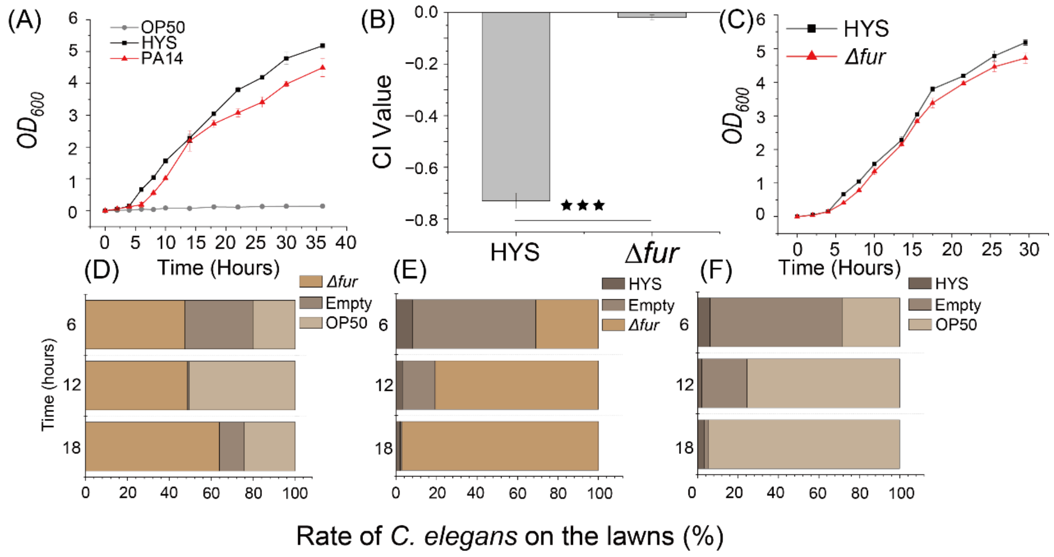

Figure S1 Growth curves of pathogens and selective avoidance of different strains by *C. elegans*. (A) Growth curves of OP50, HYS, and PA14 strains on NGM plates, Data were subjected to three independent replicate experiments and are shown as means with standard deviations. (B) The bar graph shows the usability of HYS and*Δfur* for *C. elegans*, that is, the Competition Index value. Each data is tested by three independent repeated experiments, and the average value is shown, ***$p_{adjusted}$<0.001. (C) Growth curves of HYS, and *Δfur* strains on NGM plates, Data were subjected to three independent replicate experiments and are shown as means with standard deviations. (D-F) Bar graphs show the distribution of nematodes at 6,12, and 18 h between HYS, *Δfur*, and OP50 lawns, respectively. Data were averaged from three independent replicates.

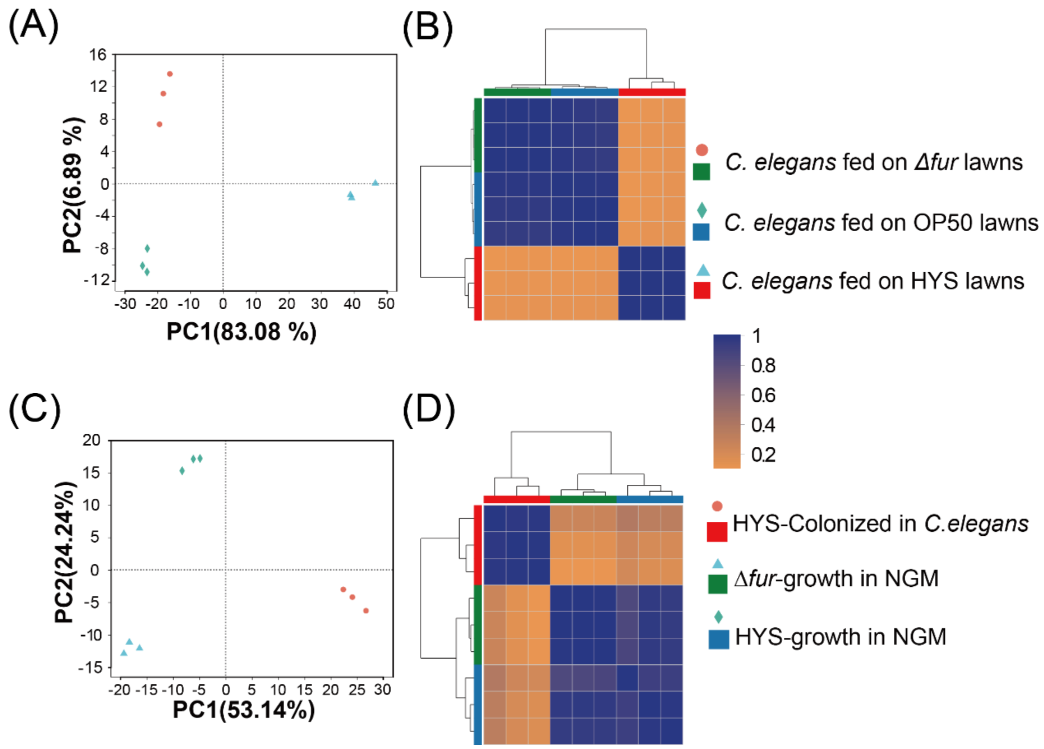

Figure S2 Biological duplications between individual samples in the transcriptome and the correlation situation. (A) PCA analysis of *C. elegans* fed on HYS, *Δfur* and OP50 gene expression data, with PC1 (83.08%) on the abs ordinate and PC2 (6.98%) on the ordinate, and orange circles indicate genes from *C. elegans* fed on *Δfur* samples, Blue triangles represent genes from *C. elegans* fed on HYS samples, and green diamonds represent genes from *C. elegans* fed on OP50 samples.(B) Cluster analysis of gene expression, with the color bar representing the level of correlation, where blue indicates high correlation and yellow indicates low correlation. In the plot, red squares represent samples of *C. elegans* fed with HYS, blue squares represent samples of *C. elegans* fed with OP50, and green squares represent samples of *C. elegans* fed with *Δfur*. (C)PCA analysis of HYS-Colonized in *C. elegans*, HYS-growth in NGM and *Δfur*-growth in NGM gene expression data, with PC1 (53.14%) on the abs ordinate and PC2 (24.24%) on the ordinate, and orange circles indicate genes from HYS-Colonized in *C. elegans* samples, Blue triangles represent genes from *Δfur*-growth in NGM samples, and green diamonds represent genes from HYS-growth in NGM samples. (D)Cluster analysis of gene expression, with the color bar representing the level of correlation, where blue indicates high correlation and yellow indicates low correlation. In the plot, red squares represent samples of HYS-Colonized in *C. elegans* samples, blue squares represent samples of *C. elegans* fed with HYS-growth in NGM, and green squares represent samples of *C. elegans* fed with *Δfur*-growth in NGM.
